# Quantitative magnetic resonance imaging (qMRI) in axial spondyloarthritis

**DOI:** 10.1259/bjr.20220675

**Published:** 2023-01-25

**Authors:** Natasha Thorley, Alexis Jones, Coziana Ciurtin, Madhura Castelino, Alan Bainbridge, Maaz Abbasi, Stuart Taylor, Hui Zhang, Margaret A. Hall-Craggs, Timothy J.P. Bray

**Affiliations:** 1 Imaging Department, University College London Hospitals NHS Foundation Trust, London, United Kingdom; 2 Department of Rheumatology, University College London Hospitals NHS Foundation Trust, London, United Kingdom; 3 Department of Medical Physics, University College London Hospitals, London, United Kingdom; 4 Centre for Medical Imaging (CMI), University College London, London, United Kingdom; 5 Department of Computer Science and Centre for Medical Image Computing, University College London, London, United Kingdom

## Abstract

Imaging, and particularly MRI, plays a crucial role in the assessment of inflammation in rheumatic disease, and forms a core component of the diagnostic pathway in axial spondyloarthritis. However, conventional imaging techniques are limited by image contrast being non-specific to inflammation and a reliance on subjective, qualitative reader interpretation. Quantitative MRI methods offer scope to address these limitations and improve our ability to accurately and precisely detect and characterise inflammation, potentially facilitating a more personalised approach to management. Here, we review quantitative MRI methods and emerging quantitative imaging biomarkers for imaging inflammation in axial spondyloarthritis. We discuss the potential benefits as well as the practical considerations that must be addressed in the movement toward clinical translation of quantitative imaging biomarkers.

## Introduction

Spondyloarthritis (SpA) is an umbrella term encompassing a heterogeneous group of inflammatory spinal disorders that share genetic, immunological, clinical, and imaging features. They are one of the most common inflammatory rheumatic disorders with an estimated prevalence of 0.1–1.4%.^1^ Axial spondyloarthritis (axSpA) refers to a subgroup characterised by **inflammation** and subsequent **structural damage** of the axial skeleton associated with symptoms of chronic back pain and sacroiliitis. AxSpA commonly affects young people and is associated with poor long-term health outcomes, exacerbated by poor mental health and difficulty with work and social function.^2,3^

There have been dramatic recent improvements in the outcomes of axSpA following the introduction of biologic therapies (which target specific inflammatory pathways to reduce inflammation),^4^ however, the efficacy of these treatments relies on effective methods for diagnosis and disease monitoring. In particular, early diagnosis is essential to prevent the accumulation of irreversible structural damage.^5^ Furthermore, biologic therapies are expensive and are associated with increased risk of serious infections as well as infusion and allergic reactions. Not all patients will respond to biologic treatment and, due to their mechanism of action, they are only likely to be of benefit in patients with active inflammatory disease. Clinical features of active inflammation are often non-specific and current disease activity measures (BATH indices)^6^ rely on patient reported measures of pain and fatigue, which are subjective and may be distorted by concomitant symptoms of chronic pain. Consequently, there is a need for improved characterisation, diagnosis and monitoring of axSpA to facilitate a more personalised approach to management and ultimately optimise patient outcomes.

Imaging modalities such as MRI and ultrasound, have emerged as valuable imaging tools for detecting early signs of inflammation, including synovitis and bone marrow oedema.^5^ MRI now forms a core component of the diagnostic pathway in axSpA^7^ and there is good evidence that the signal abnormalities detected on MRI are linked to inflammation detected histologically.^8^

However, despite achieving wide clinical uptake, imaging techniques such as conventional MRI and ultrasound are limited by: (i) the fact that the observations are not specific to the disease process in question and can be ‘confounded’ by other processes, and (ii) their reliance on qualitatively described information which is interpreted subjectively depending on the experience and expertise of the reader, and the clinical information provided. As a result, the same patient undergoing the same scan could potentially receive varying therapeutic management, depending on scan interpretation.^9,10^ Moreover, variations in image properties and contrast may also occur due to different MRI hardware, acquisition parameters and imaging protocols^11,12^ (even when the protocols are nominally the same) which can potentially affect subjective interpretation and comparisons. These problems contribute to difficulty with monitoring inflammation over time and adjusting treatment accordingly in patients with longstanding disease.

As a result of these limitations, there has been interest in the development of quantitative MRI (qMRI) techniques. The use of qMRI techniques offers a potentially more objective and reproducible approach to image analysis/interpretation which minimises subjectivity, can disentangle the effects of pathology from other processes and could enable precise measurement of changes over time. In clinical practice, qMRI techniques could potentially provide a more robust assessment of disease activity and response to treatment, facilitating a personalised management approach. In clinical trials, qMRI techniques could offer accurate and objective measures of disease activity which do not rely on potentially variable, subjective patient reported outcome measures.

## What is qMRI?

qMRI has been defined as “the extraction of a characteristic from an MR image that has a magnitude that can be expressed as a number with units”.^13^ Whereas the contrast in conventional MRI [*e.g.* the widely-used short inversion time inversion recovery (STIR) sequence] is influenced not only by the ‘target’ process (inflammation) but also by other factors (including hardware, tissue characteristics, acquisition, reconstruction and display of greyscale).^14^ qMRI enables us to tease out and measure the direct contribution of each process.

**Figure 1.**
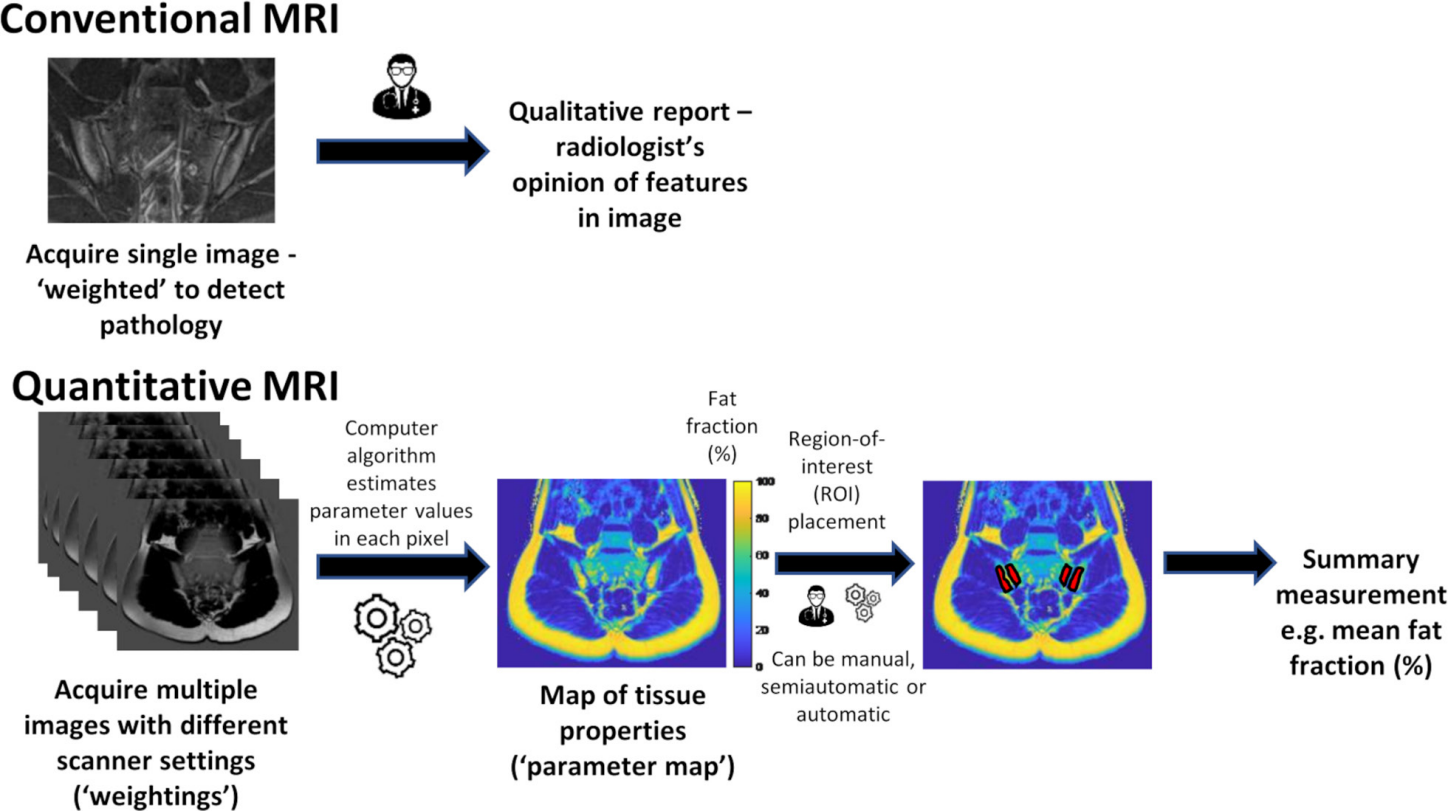
A flowchart of the steps involved in the qMRI pipeline (with chemical shift encoded MRI (CSE-MRI) as an example) and comparison with conventional MRI acquisition.

To do this, qMRI incorporates a succession of multiple images acquired with different scanner settings (these settings might include, *e.g.* repetition time, echo time or b-value) which enables analysis of a range of different tissue characteristics, such as relaxation properties (*e.g. T*_1_, *T*_2_, *T*_2_*), fat content or diffusivity, respectively. For example, in T_2_ relaxometry echo time would be incrementally increased, while keeping all other parameters the same. Each of the images in a series can be regarded as weighted by the property of interest. This may require administration of an external agent, *e.g.* intravenous injection of a tracer or contrast agent, or rely on native tissue characteristics. Figure 1 demonstrates a flowchart of the steps involved in the qMRI pipeline.

A computer model is then developed to relate the underlying (patho)physiological processes of interest to the data acquired by imaging. This computer model is used to estimate tissue *parameters* representing the (patho)physiological process of interest in each voxel. A map of the chosen parameter is produced where each voxel has a measurable numerical value that reflects the intrinsic properties of a tissue. Once parameter maps have been generated, a metric of disease must be extracted which is typically achieved by region of interest (ROI) placement, whereby abnormal regions are manually or automatically *segmented*. Once the ROIs have been placed, summary metrics can be extracted using a variety of statistical methods, the simplest of which is simple averaging over the voxels in the ROI.

The summary metrics produced by this pipeline can be regarded as quantitative imaging biomarkers (QIBs). QIBs are defined by Sullivan et al^15^ as “objectively measured characteristics derived from an *in vivo* image as indicators of normal biological processes, pathogenic processes, or response to a therapeutic intervention”. It is worth noting this definition can include QIBs which are not generated from qMRI, *e.g.* those generated from the segmentation of conventional MR images without the need for parameter maps (*e.g.* measurement of lesion volume). Potential applications of QIBs include screening for disease, diagnosing and assessing disease severity, and monitoring disease activity and therapeutic response.^16^ QIBs may also be valuable in the development of novel therapeutic agents, where they offer specific, quantifiable measures of particular biological processes of interest.^16^

This review aims to summarise the current research on qMRI methods in axSpA and to discuss the practical considerations that must be addressed before qMRI techniques are translated into clinical practice.

## Quantitative MRI techniques and biomarker development in axSpA

A number of different qMRI techniques have been explored in imaging axSpA. The QIBs they provide can be designed to target two broad pathophysiological processes of interest: (i) **inflammation**—the initial response to a harmful stimulus, typified by cellular infiltration, increased extracellular fluid, increasing vascularity and replacement of the normal fatty marrow in subchondral bone (producing signal abnormalities referred to as bone marrow oedema (BMO) on conventional MRI scans),^17^ and (ii) **structural damage**—defined as loss of structural or functional integrity of tissue occurring as a result of inflammation, and typified by subchondral fat metaplasia, sclerosis, joint erosions and eventual joint fusion.^17^ The presence of BMO, fat metaplasia and erosions in the sacroiliac joints (SIJs) contribute to the diagnosis of axSpA^18^ and have prognostic significance.^19^ qMRI methods in axSpA might be designed therefore to detect and characterise both acute inflammation and structural damage, and ultimately aim to use this information to guide management on an individualised basis. A summary of qMRI techniques and the pathophysiological process(es) they are designed to target is described in Table 1.

**Table 1.** A summary of qMRI techniques used in axSpA imaging

qMRI technique	Biomarker	Target pathophysiological process(es)	Pros	Cons
**DWI**	ADC	BMO	High availability	Low spatial resolution; low SNR; requires effective fat suppression
	**IVIM:** Pure diffusion (Ds) Perfusion fraction (ƒ) Pseudodiffusion coefficient (Df)	BMO (Ds) Increased perfusion/vascularity (ƒ,Df)	Non-invasive measure of dynamic properties	Less availability
**Relaxometry**	T_2_	BMO or cartilage damage	High availability; high spatial resolution	Susceptible to external confounding factors
	T_1_	BMO		
	T_2_*	BMO		
**CSE-MRI**	PDFF	BMO or fat metaplasia	High spatial resolution	Lower sensitivity to tissue properties
**DCE-MRI**	Pharmacokinetic perfusion parameters, *e.g.* signal intensity curve, RE, ME, TTP, and BE	Increased perfusion/vascularity	Measures dynamic properties	Invasive

ADC, apparent diffusion coefficient; BE, brevity of enhancement; BMO, bone marrow oedema; CSE-MRI, chemical shift encoded MRI; DCE-MRI, dynamic contrast enhanced MRI; DWI, diffusion-weighted imaging; IVIM, intravoxel incoherentmotion; ME, maximum enhancement; PDFF, proton density fat fraction; RE, relative enhancement; SNR, signal-to-noise ratio;TTP, time to peak.

### Diffusion-weighted imaging

**Figure 2.**
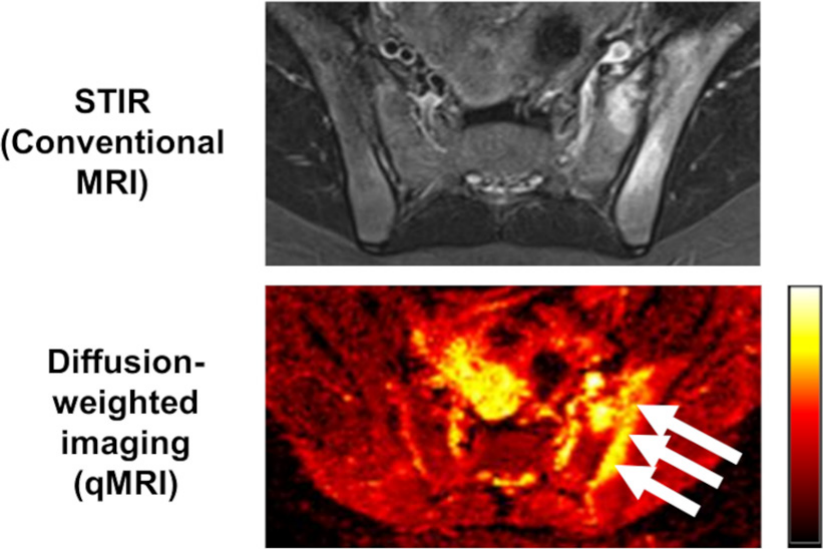
Active inflammation at the left sacroiliac joint detected on the short inversion time inversion recovery (STIR) image (top) is detectable on the qMRI diffusion-weighted image (bottom). The arrows indicate bone marrow oedema (raised apparent diffusion coefficient, ADC value).

Diffusion-weighted imaging (DWI) is a method of signal contrast generation based on differences in the freedom of water diffusion in tissue, known as diffusivity.^20^ In DWI, a series of images is acquired with different degrees of diffusion weighting. The b-value represents the degree of diffusion weighting that is applied: typically, a range of b-values are acquired including *b* = 0 (no diffusion weighting) to higher b-values (increased diffusion weighting). The signal on the higher b-value images is increasingly attenuated with greater tissue diffusivity. Tissue diffusivity itself is commonly estimated using a monoexponential signal model in which a single ‘apparent’ diffusion coefficient (ADC) value is used as a ‘summary’ measure of tissue diffusivity; this step effectively disentangles the diffusivity measurement from the raw signal intensities at the different b-values.^21^ The presence of inflammatory exudate in the bone marrow causes expansion of the extracellular space and an increase in the proportion of extracellular water molecules.^21^ Extracellular water molecules experience greater diffusivity/freedom of diffusion relative to intracellular water molecules,^20^ and consequently increase the ADC value. ADC measurements therefore offer a means to quantify expansion of the extracellular space and serve as a QIB, enabling the measurement of BMO, and thus reflecting disease activity^21^ (Figure 2).

Studies of patients with axSpA have demonstrated that ADC values are significantly higher in patients with BMO compared to controls.^22–27^ ADC values are also able to differentiate active *vs* inactive disease in axSpA.^28^ Subsequent studies have shown that ADC values decrease over time in response to infliximab^29^ and other tumour necrosis factor (TNF) inhibitors^24,30^ in patients with sacroiliitis, supporting the biological validity of ADC values as QIBs. Some studies have also shown a positive correlation between ADC values and patient-reported disease activity measures, such as the Bath Ankylosing Spondylitis Disease Activity Index (BASDAI).^27,31^ Although the relationship is generally weak, this is likely reflective of the multifactorial nature of pain aetiology and perception, and the fact that symptoms and imaging may represent different biological processes. While ADC measurements provide a useful summary measure of tissue diffusion, more sophisticated models of diffusion such as intravoxel incoherent motion (IVIM), can separate the contributions of tissue diffusivity from the effect of perfusion^32–35^ and may provide a more comprehensive description of the MRI signal than standard monoexponential models.^35^

A limitation of DWI is its relatively low spatial resolution and signal-to-noise ratio (SNR) in the bone marrow, which limits its ability to detect small or low-grade areas of inflammation.

### MR relaxometry

MR relaxometry is a method for measuring T_1_, T_2_ or T_2_* relaxation times from MR images^11^ which produces images that represent the spatial distribution of relaxation times known as T_1_, T_2_ and T_2_* maps.^11^ In axSpA, T_2_ mapping has been the most commonly used MR relaxometry technique. T_2_ mapping is obtained by the measurement of T_2_ relaxation times after acquiring multiple images with different echo times, and provides information on water molecular movement (specifically the molecular tumbling rate) in tissue.^36^ In the simplest case, a monoexponential single model is fitted to the data which enable the T_2_ relaxation constant for each pixel to be obtained.^37^

In spondyloarthritis, T_2_ mapping techniques have focused on the subchondral bone marrow or articular cartilage of the SIJs.^37^ T_2_ values increase as a consequence of increased tissue water content and can therefore detect BMO or early cartilage damage, as a result, T_2_ mapping of the SIJ subchondral bone^28^ and cartilage^38,39^ can be used to differentiate patients with axSpA from controls. Francavilla et al^40^ employed the use of T_2_ mapping of SIJ cartilage in children and young adults with sacroiliitis which indicated^39^ a trend toward increased T_2_ relaxation time in patients with axSpA, however, the relationship was not statistically significant, likely due to the limited sample size (only 14 subjects).

A recent pilot study by Lin et al^41^ compared different MR relaxometry techniques (T_1_ mapping, T_2_ mapping, T_2_* mapping) for mapping the subchondral bone marrow and demonstrated that these could be used to monitor response to TNF inhibitor treatment. Lin et al^41^ found T_1_ mapping had better diagnostic efficacy than T_2_ mapping which was susceptible to confounding effects of the external environment including age, bone marrow composition, fat deposition and changes in the structure of bone trabeculae.

A study by Wang et al^28^ which compared T_2_ mapping to DWI in patients with active sacroiliitis, inactive sacroiliitis and controls, found ADC values had a higher diagnostic efficacy for axSpA than T_2_ values.

### Chemical shift-encoded MRI

Chemical shift-encoded MRI (CSE-MRI) is an imaging technique for differentiating and quantifying the signals from fat and water in individual voxels. It exploits the difference between the precession frequencies of protons in fat and water molecules, which depends on subtle variations in the local magnetic field experienced by these protons.^42^ Fat molecules have higher shielding of protons in the electronic nucleus than water molecules, so protons in fat molecules precess at a slightly lower frequency.^42^ This change in precession frequency is known as chemical *shift*. As the MR signal is sampled at multiple timepoints, the signal from a voxel can be separated into water and fat components. The proton density fat fraction (PDFF) refers to the proportion of the total signal (S) arising from fat [*i.e.* FF = S_fat_/(S_water_+S_fat_)] once corrected for confounding factors such as T_2_* and the spectral complexity of fat.^43^ This means that the PDFF can estimate the density of fat in the tissue independent of the scanner and acquisition parameters.^21^

**Figure 3.**
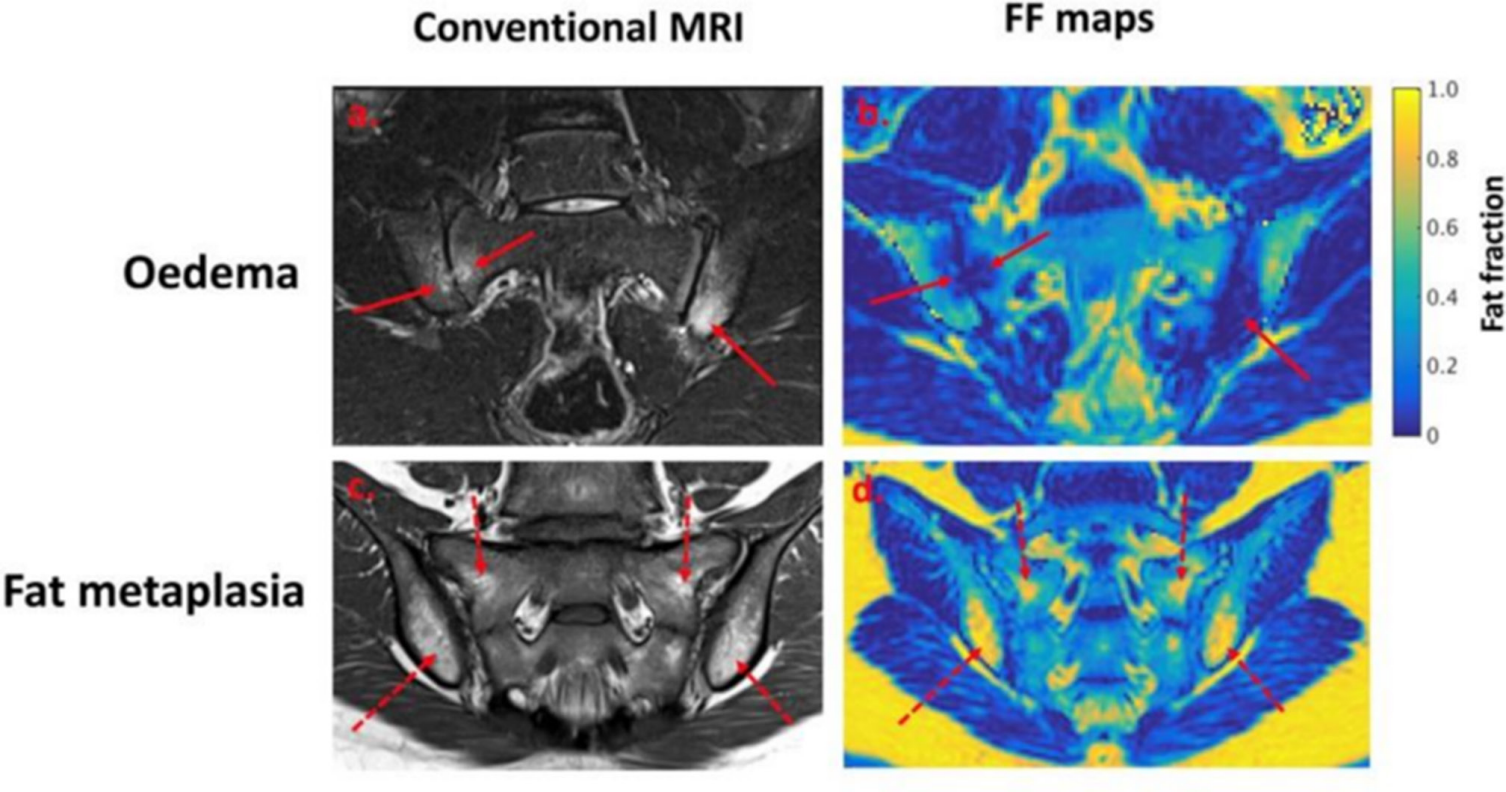
Fat fraction (FF) mapping detects active inflammation and bone marrow oedema (figure reproduced from Bray et al^44^). Active inflammation detected on the STIR image (**a**) is detectable on the fat fraction map as a reduction in fat fraction (**b**). The fat fraction map can also detect fat metaplasia (**d**), a form of structural damage which is conventionally identified using T1 weighted images (**c**).

CSE-MRI/PDFF imaging in young patients with axSpA has shown that PDFF measurements are reduced in areas of BMO through a reduction in the normal marrow fat content (Figure 3).^44^ PDFF measurements can also be used to identify structural damage such as the characteristic periarticular fat metaplasia, which produces an increase in fat fraction, using the same acquisition (Figure 3).^44–47^ PDFF measurements are thought to undergo biphasic changes over time, with initial PDFF reductions in areas of inflammation followed by PDFF increases (beyond normal values) due increased osteoclastic activity. The ability of PDFF imaging to detect structural damage has been used to distinguish patients with inactive sacroiliitis from healthy controls,^26^ thus PDFF measurements could serve as QIBs to monitor disease progression over time.^47^ CSE-MRI is promising in its potential to quantify both active and chronic inflammation in a single acquisition. It can also be combined with techniques such as T_2_ mapping to enable multiparametric inflammation assessment.^48,49^

### Dynamic contrast-enhanced imaging

**Figure 4.**
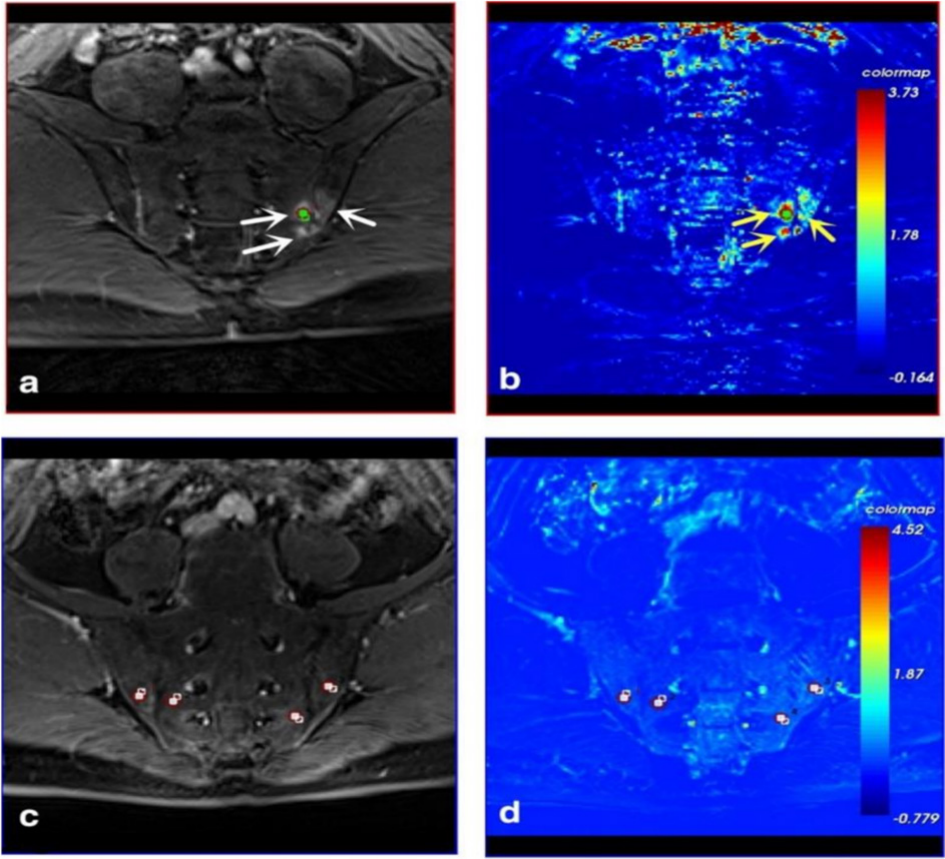
DCE-MRI of two patients with axSpA demonstrating active (**a,b**) and inactive (**c,d**) disease (figure reproduced from Zhang et al^50^). (**a**) active inflammation is detected as an area of enhancement (arrows) in the right SIJ on the contrast-enhanced T1-weighted MR image. (**b**) K^trans^ (forward volume transfer constant) colour map. K^trans^ is a measure of capillary permeability which is calculated by measuring the accumulation of contrast in the extravascular-extracellular space. The map shows high K^trans^ values (K^trans^  = 1.975) in the inflamed right SIJ (arrows). (**c**) No significant enhancement on the enhanced *T*1 image. (**d**) K^trans^ values were relatively low in bilateral sacroiliac joint; multiple ROIs were placed on the articular surface and the average K^trans^ was 0.388.

**Figure 5.**
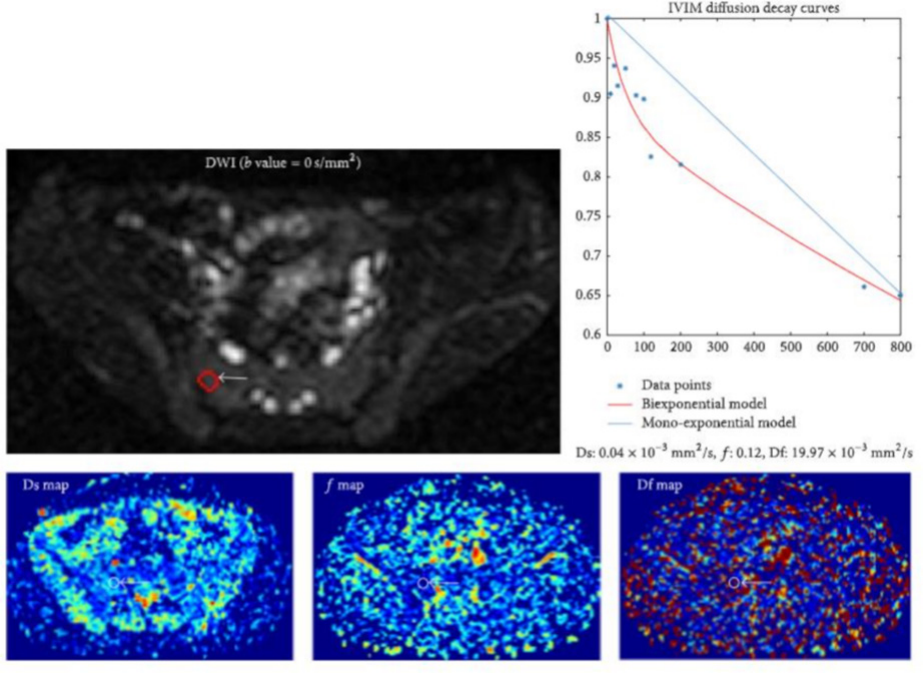
IVIM DWI in a patient with axSpA (figure reproduced from Zhao et al^52^). On the Ds (pure molecular diffusion) map, ƒ (perfusion fraction) map, and Df (perfusion-related diffusion) map, ROIs are placed in the juxta-articular bone marrow. IVIM diffusion decay curves can be plotted with b value (x axis) against log plot of signal intensity (y axis) based on a monoexponential model (blue line) and biexponential model (red line). For this ROI, IVIM DWI signal intensity decay shows a nonlinear relationship (top right).

Dynamic contrast-enhanced imaging (DCE-MRI) is a method for measuring tissue perfusion following the administration of an intravenous gadolinium-based contrast agent.^50^ Multiple acquisitions are performed in rapid succession and the temporal changes in signal intensity are analysed (Figure 4). A variety of kinetic parameters can be calculated from DCE-MRI, such as those described by Tofts et al.^51^ Alternatively, signal intensity curves of contrast enhancement over time can be analysed (known as the heuristic method), with several distinct curve patterns being described.^21^ Quantitative DCE-MRI parameters can differentiate between active and inactive sacroiliitis, likely due to increased perfusion, destruction of microvascular architecture and increased capillary permeability in active inflammation.^50^ Furthermore, DCE-MRI can monitor changes in inflammation with biologic therapy.^29^ A potential limitation of DCE-MRI in clinical practice is the requirement for technical consistency, particularly in contrast administration and acquisition timings following the bolus. Zhao et al^52^ demonstrated the potential of IVIM diffusion models as an alternative method to DCE-MRI for acquiring perfusion parameters in axSpA which negates the need for invasive contrast agents (Figure 5).

## Moving towards clinical translation: practical considerations

Although the techniques discussed show promise, most remain at an early stage of development in their application to axSpA and to date, their usage is sporadic. To achieve more widespread adoption of these methods into routine clinical practice several important research steps need to be undertaken. Here, we discuss some of the key practical considerations in the path towards clinical implementation.

### Selecting a qMRI method

Acquiring qMRI images is time consuming, therefore a key practical consideration is selecting the most appropriate qMRI method to explore for further development. This will depend on the ability of the candidate method to target the underlying pathophysiological process of interest and its availability. DWI has been extremely successful for neurological and oncological imaging, and its wide availability has arguably been a major driver for its investigation in rheumatological diseases. However, the use of DWI in imaging of inflammation is debatable. In particular, since areas of oedema can be small, the limited spatial resolution of DWI scans is a potential obstacle to its success. Moreover, in tissues containing a substantial proportion of fat (such as bone marrow), the robustness and reproducibility of ADC measurements depends on effective fat suppression. In addition to the variable quality of fat suppression, the use of different pulse sequences and varying diffusion weighting across scanners also contributes to the variation in ADC measurements in the bone marrow,^53^ providing a potential barrier to translation. Nonetheless, from a pathophysiology perspective DWI can capture the changes in the extracellular space and/or cellular infiltration in areas of BMO, and further technical developments in DWI methodology may help to mitigate these issues. Other methods such as CSE-MRI offer much higher spatial resolution with volumetric acquisitions, but may lack the intrinsic sensitivity to tissue properties provided by conventional methods such as STIR and by quantitative methods such as DWI. A potential compromise is to use methods combining chemical shift encoding with T_2_ mapping, such as that proposed by Gollifer et al.^49^ This method remains at an early stage of development but promises an opportunity to combine the benefits of CSE-MRI with sensitivity to tissue water properties.

### Reproducibility of the qMRI method

Another practical consideration is demonstrating the reproducibility of the chosen qMRI method. Reproducibility is a measurement of precision that occurs under differing conditions such as different operator, equipment, software and location^15^ and is a key component in establishing the technical validity of the qMRI method. For example, a reproducibility study might involve evaluating the qMRI method on different scanners made by different manufacturers, and including systems with different field strengths, with the goal of assessing the performance of the QIB across these systems.^54^ One of the challenges in establishing reproducibility is the large amount of variability in image acquisition and post-processing techniques in clinical imaging.^13,16^ Although imaging equipment of differing manufacturers and models produce images that are comparable for most diagnostic radiology purposes, they often have important differences that affect imaging biomarker (IB) acquisition and quantification, and subsequently impact on reproducibility.^16^ For the purpose of technical validation, reference materials and objects (digital or physical phantoms) provide a valuable means to evaluate the performance of the method in terms of bias.^15^ Fat–water phantoms can also be scanned across multiple scanners and at different sites, allowing evaluation of reproducibility.^46^ The results can be used to refine, adjust, or standardise protocols to improve reproducibility metrics.

Bainbridge et al^46^ demonstrated the reproducibility of PDFF measurements across different centres in healthy volunteers with a view to its use in spondyloarthritis.^46^ Using fat–water phantoms, Hernando et al^54^ also demonstrated the reproducibility and accuracy of PDFF measurements across centres, vendors, field strengths and protocols. However, the reproducibility of ADC measurements is a known limitation of DWI^53^ and to our knowledge, there are currently no studies addressing this issue in the context of spondyloarthritis.

### Measurement extraction

Developing a method for measurement extraction is a further consideration in the development of a qMRI workflow. This can be particularly challenging in imaging inflammation as the diffuse and heterogenous nature of the underlying pathophysiological process makes it not easily amenable to simple measurements, such as size. Furthermore, in axSpA, inflammation may affect a wide anatomical area, including the SIJs and/or spine, and features of active and chronic inflammation may coexist and overlap. This means extracting meaningful measurements from the images is challenging.

**Figure 6.**
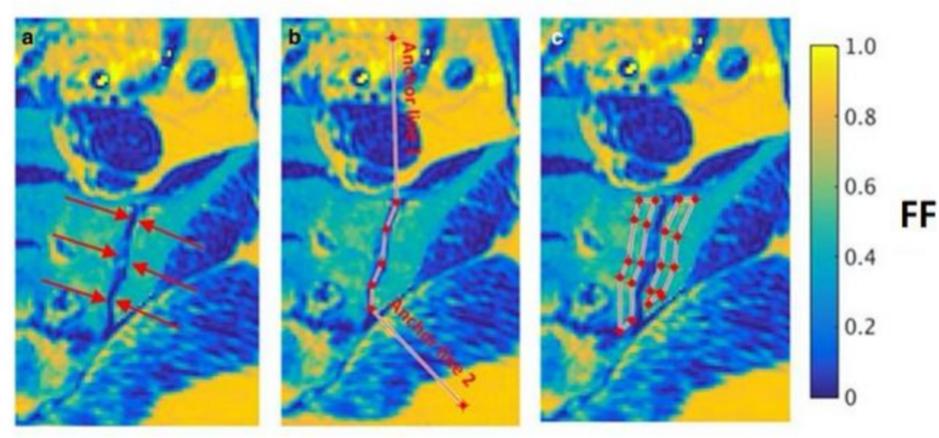
Improving the objectivity of ROI placement using the BEACH tool (figure reproduced from Bray et al^45^ (with fat fraction (FF) maps as an example). With this tool, the user identifies the joint line (**a**) and places a linear region of interest to define the joint (**b**), as well as anchor lines to define the angle made between the joint and the edge of the bone (see reference for details). The tool then automatically propagates a polygonal ROI onto the subchondral bone of interest (**c**). This region also includes normal bone, but the BEACH tool uses histographic analysis to target areas of maximal abnormality.

In homogenous tissue, extracting measurements can be obtained by simple ROI placement. However, this approach introduces the potential for subjectivity and may be inadequate in diseases where inflammation occurs heterogeneously, and has been a source of criticism in DWI.^55^ Several studies have proposed alternatives to manual ROI placement which can substantially reduce subjectivity in measurement extraction. Bray et al developed a semiautomated analysis tool known as Bone Edema and Adiposity Characterisation with Histograms.^45^ With this approach, the SIJs are manually delineated (a simple task which can be achieved with excellent agreement between observers); the software then automatically propagates ROIs onto the subchondral bone (Figure 6). Pixel values from all ROIs are then analysed histographically, thus targeting areas of maximal abnormality.

**Figure 7.**
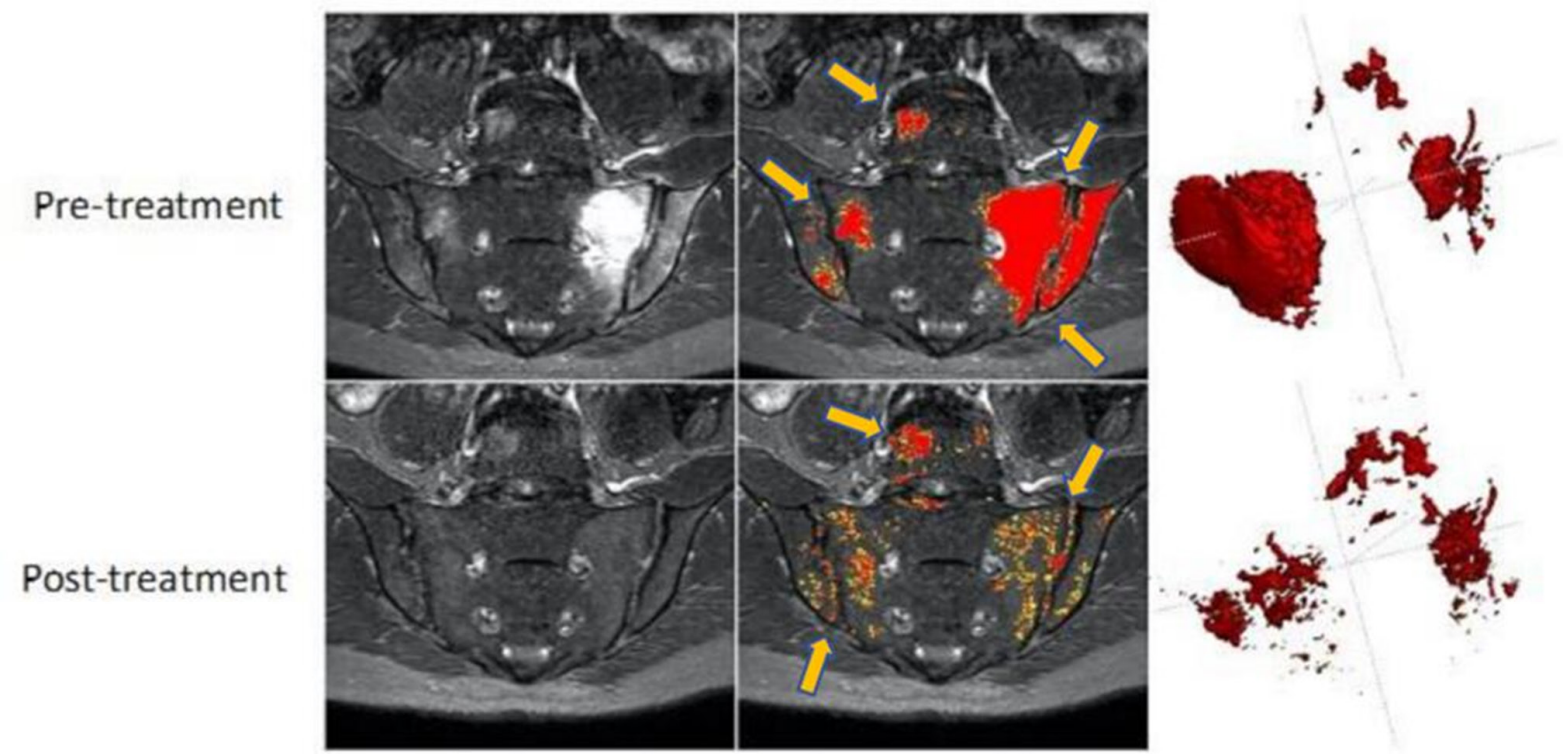
Semiautomated analysis using deep learning (figure reproduced from Hepburn et al^56^). In the method proposed by Hepburn et al,^56^ areas of inflammation (shown as hyperintense on STIR images, left column) are segmented by an algorithm combining deep learning with intensity-based thresholding (segmented disease in middle (shown by arrows) and right column). In this example, the volume of abnormal tissue is shown to markedly reduce after treatment.

Deep learning methods may also have a role in improving the objectivity of measurement. For example, Hepburn et al^56^ developed a semi-automated, AI-based method which labels inflammatory lesions in patients with axSpA using intensity-based thresholds derived from comparison with normal marrow (Figure 7). This method yielded substantially higher intraobserver agreement compared to manual delineation (Dice coefficient 0.84 *vs* 0.55, respectively).^56^ A different AI-assisted method described by Faleiros et al^57^ demonstrated the potential of machine learning models to aid the classification of SIJ MRIs as positive or negative for active inflammation with 100% sensitivity, 96% specificity and 85% accuracy. Although these AI-assisted methods have only been applied to conventional MRI images thus far, application to qMRI should be straightforward and offers a promising and consistent method for extracting qMRI data.

### Lack of a readily attainable reference standard

A further practical consideration is the lack of a readily available, robust reference standard in axSpA which makes biologic validation of QIBs arguably more challenging. Histological evidence of inflammation is often not attainable and, even when available is subject to sampling bias and does not necessarily provide an adequate reference standard against which to validate the complex, spatially distributed changes identified by imaging. Some studies have correlated conventional imaging with histology, but this has not been repeated for novel qMRI biomarkers.^8,58,59^ Alternative clinical indices, symptom scores and blood markers, are often non-specific and have not demonstrated consistent correlations with disease activity on MRI.^27,60^ Clinical assessment and diagnosis are therefore often relied upon as a reference standard,^61^ although this may not be a practical approach in early phase studies. As a result, most studies have used indirect means for initial biomarker validation, typically demonstrating an association between signal abnormalities on conventional MRI and parameter abnormalities on qMRI.^22,29,30,62^ Although imperfect, this approach offers a ‘sense check’ ensuring that the expected effect is present, and can enable estimation and comparison of the effect size for different QIBs before larger, more resource-intensive studies are performed.

## Future directions

Quantitative imaging techniques and biomarkers offer significant potential in the ability to individually assess bone marrow characteristics, potentially allowing for more robust and accurate measures of disease activity in rheumatic disease. However, to date, none of the techniques described has reached wide-scale clinical use. We suggest that careful choice and tailoring of qMRI methods to the target inflammatory process and improved strategies for measurement extraction may help to increase the chance of future successful translation of candidate inflammatory QIBs. The development of AI-based techniques for automated measurement extraction could facilitate efficient, consistent, and objective assessment of qualitative images. However, the development of these AI-based algorithms will require large data sets, meaning that collaboration and data-sharing across different centres will be crucial. Furthermore, large-scale, multicentre trials will ultimately be needed to demonstrate the reproducibility, clinical efficacy and cost effectiveness of candidate qMRI methods.

It is important to appreciate that multidisciplinary collaboration is essential at each stage of QIB development and validation to increase the chances of translational success. Expertise across radiology, rheumatology, medical physics, computer science, and statistics is paramount. Collaboration with the pharmaceutical industry could enable incorporation of QIBs as endpoints in clinical trials and this might help to improve power and/or reduce sample size, as well as enable exploration of novel therapeutic mechanisms (*e.g.* drugs which specifically aim to prevent new bone formation). Ultimately, the implementation of quantitative techniques into clinical practice will require the support and engagement of the multidisciplinary team involved in patient care.

## Conclusion

QIBs have the potential to inform clinical decision-making in inflammatory rheumatic diseases by providing more precise, accurate and individualised assessment of bone marrow characteristics. Although there have been significant recent advances in the quantitative imaging of inflammation with many promising emerging biomarkers, there are a number of practical considerations that must be addressed to facilitate clinical translation. We suggest that further work should focus on tailoring qMRI methods to the target pathology and improving methods for measurement extraction.
